# Rethinking ethical reflexivity and oversight in health research through an ecosystem approach: A workshop report

**DOI:** 10.1177/17470161251346253

**Published:** 2025-09-12

**Authors:** Katharine Wright, Joseph Ali, Caesar Atuire, Phaik Yeong Cheah, Anna Chiumento, Agata Ferretti, Adrienne Hunt, Sharon Kaur, Rachel L. Knowles, Carleigh B. Krubiner, Florencia Luna, Paul Ndebele, Ana Palmero, James Shaw, Effy Vayena, Teck Chuan Voo, Jantina de Vries, Katherine Littler

**Affiliations:** 1 World Health Organization, Geneva, Switzerland; 2 Johns Hopkins Berman Institute of Bioethics, Baltimore, MD, USA; 3 Johns Hopkins Bloomberg School of Public Health, Baltimore, MD, USA; 4 University of Ghana, Accra, Ghana; 5 University of Oxford, UK; 6 Mahidol University, Bangkok, Thailand; 7 University of Edinburgh, UK; 8 ETH Zurich, Switzerland; 9 Universiti Malaya, Kuala Lumpur, Malaysia; 10 Medical Research Council, UK Research and Innovation, Swindon, UK; 11 Wellcome Trust, London, UK; 12 FLACSO, CABA, Argentina; 13 CONICET National Scientific and Technical Research Council, CABA, Argentina; 14 The George Washington University, DC, USA; 15 University of Toronto, Canada; 16 SingHealth, Singapore, Singapore; 17 University of Cape Town, South Africa

**Keywords:** ethics ecosystem, ethics oversight, ethical reflexivity, ethics review, REC review, research ethics

## Abstract

As the scope of morally relevant considerations widens and new challenges emerge at the frontiers of health innovation, there are questions about the appropriate role and remit for research ethics review, within the broader context of the whole health research ecosystem. Drawing on discussion at a satellite meeting at the 2022 Global Forum on Bioethics in Research in Cape Town, we argue that the ethical conduct of research is the responsibility of *all* stakeholders in the research ecosystem – from funders, governments and research institutions to individual research teams and ethics committees. As a research community we need to espouse, and take action to achieve, more distributed approaches to ethical scrutiny and reflexivity. A crucial element of such a shift should be the development of collaborative and non-adversarial relationships between researchers and ethics committees that recognise and respect the mutual responsibilities of all parties to promote ethical research conduct. In tandem with the development of systems to support the exercise of ethical responsibilities across the research ecosystem, committees need to reconceptualise their role, in partnership with communities, as one of providing accountability through a focus on how research promotes participant agency and the common good.

## Introduction

Over the last 50 years, great strides have been made in formalising the ethical oversight of research in the health sector through the development of systems of independent ethics review – largely driven by the motivation to protect human research participants from potential harms and exploitation. However, in recent decades we have seen an evolution both in the types of research being conducted, and in our conceptions of what ‘ethical’ research entails. The health research sector is experiencing a period of rapid change, developing new kinds of studies and using ever-more complex methodologies, such as adaptive platform trials (Singh, 2023). There is an overlap between health-related research and data-science with the rise of big data and the role of Artificial Intelligence (AI) in health and healthcare (Ferretti et al., 2020), and growing attention to data-related ethical issues including the ever-increasing collection of sensitive data and individual return of results (Ochieng et al., 2021). Research itself is becoming increasingly international and interdisciplinary, with questions about the equity of partnership arrangements in international collaborations a focus of ethical concern (Faure et al., 2021; Horn et al., 2023; Kalinga, 2019; Parker and Kingori, 2016).

As the scope of morally relevant considerations widens and new challenges emerge at the frontiers of health innovation, there are increasing questions about the appropriate role and remit for research ethics review within the broader context of the health research ecosystem (London, 2022; Simpson, 2011; Zwi et al., 2006). The WHO Health Ethics and Governance Unit convened a satellite workshop at the Global Forum on Bioethics in Research in Cape Town in December 2022 (Global Forum on Bioethics in Research (GFBR), 2022) to explore these questions, involving 19 leading ethicists^1^ from across the globe with experience of ethical review systems across all six WHO regions. In this article, we refrain from re-rehearsing the history of the emergence of research ethics, which existing literature comprehensively addresses (e.g. Emanuel et al., 2008; Fischer, 2006), to focus instead on the core of the workshop discussions: what might be the key ingredients of an ecosystem model of research ethics, and what implications might this model hold for current systems of ethical review? Our discussions drew on participants’ extensive knowledge of scholarship in this field, referenced where appropriate in our account below. Importantly, they also drew on participants’ familiarity with the practical challenges of achieving both meaningful and proportionate ethical oversight of health research in many different countries and contexts. Our intention is to stimulate engagement and debate among the wider research ethics community on the dispersal of ethical responsibilities across the research ecosystem, and in so doing to re-think the scope of expectations that are placed on ethical review.

## Limitations of current approaches to ethical oversight of research

While independent research ethics review has a critical oversight role to play in ensuring that health research is conducted ethically, it cannot and should not be held responsible for tending to the full range of ethical considerations arising in the health research landscape. In addition to the developments in research methodologies and ethical analysis outlined above, there are longstanding practical and substantive reasons why we must revisit the appropriate scope and function of ethics review committees.^2^ These include widespread misperceptions among researchers as to the role of ethics review, timing constraints with review offering only static snapshots in time, regulatory capture with ethics review risking conflation with compliance and the limitations of a ‘case-by-case’ approach to review. We have outlined these in Box 1 to orient and signpost the reader to wider discussion of the challenges and critiques that ethics committees currently face, and which prompted the workshop to focus on the importance of articulating an ecosystem approach, for which this article aims to chart a roadmap.

Workshop participants agreed that it is not only impossible to expect a system of oversight, set up to review studies on a case-by-case basis, to carry responsibility for broader ethical considerations such as equity within the health research system and contribution to global public goods: it would also be undesirable. To do so would divert focus from the many other actors within the health research system who have greater influence and responsibilities for shaping these wider ethically-relevant impacts, including funders, research teams and their employing organisations, community advisory boards, journals, governments and increasingly, advocacy groups and private sector actors. We need to think afresh about the nature of the ethical responsibilities of these multiple parts of the research ecosystem; of how these dispersed responsibilities should be exercised; and then of the role within this ecosystem of ethics review committees. This is the purpose of this article, to advance an ecosystem model for ethics by drawing together evolving research and practical strategies that support dispersed responsibilities for promoting ethical research conduct for others to take-up and implement within their own systems and structures.

## The overarching aim: Achieving ethically commendable research

It was strongly argued at the workshop that an exploration of new approaches to ethical reflection on, and scrutiny of, health research should not be restricted to the question of ‘how should ethics committees operate?’ but rather begin with the much more open question: ‘What is needed for us to achieve ethically commendable research?’ In other words, how do our existing processes and systems across the many stakeholders and stages of the research ecosystem need to change so that they are actively concerned not only with preventing unethical research practices, but also with actively supporting and enabling scientific progress in pursuit of the common good? From this starting point and in the context of the responsibilities of multiple other stakeholders, what should this mean for ethics committees themselves?

Starting with the first question of the pursuit of the common good, we recognise that there is much debate to be had about what, in particular contexts, constitutes the common good, not least with reference to the question of ‘good for whom’. Recognising that here we can only touch upon these debates, we suggest that an excellent starting point in the context of health research is that of ‘the egalitarian research imperative’ championed by Alex John London, according to which: ‘the public purpose of research is to generate the knowledge necessary to bridge gaps in the capacity of the basic social institutions of a community – such as its system of public health and clinical medicine – to safeguard and advance the basic interests of that community’s members’ (London, 2022: 251). In light of the increasing recognition of the critical role played in human health and well-being by the health of physical ecosystems, alongside human-made institutions, we note that further refinements of the ‘public purpose of research’ should also include explicit reference to the purpose of advancing planetary health (The Lancet Public Health, 2022).

Crucially, this reframing also makes clear that responsibility for ethically commendable research – underpinned by a common aspiration and commitment to such an aim – needs to be shared across *all* stakeholders in research: from lead researchers, other research team members, and their institutions; to ethics committees, research funders, journals, governments, and private sector actors who facilitate or engage in research. Funders, for example, make intrinsically ethical decisions in deciding how to allocate their resources, from their strategic decisions about funding priorities through to the guidance they give to those tasked with reviewing funding applications, and any limitations or requirements they may set on the use of budgets (Wright, 2020). Research institutions determine the environment in which research teams operate, and can promote (or inhibit) a culture where reflective approaches to research are supported (Joynson and Leyser, 2015; UK Research Integrity Office and ARMA, 2021). Journals exercise enormous influence over what research gets disseminated (and what does not), and through their publishing policies can actively promote factors such as fair authorship criteria (Bhakuni and Abimbola, 2021; Gurung et al., 2021; Morton et al., 2022).

In order to realise the exercise of these shared responsibilities, it may thus be helpful to think in terms of an ‘ethics ecosystem’ encompassing multiple responsibilities for ethical reflexivity and scrutiny, embedded across multiple actors and at multiple time points within the wider research ecosystem (Tackett et al., 2024). A process of independent ethics review is clearly one pillar within this ethics ecosystem, providing both an independent opportunity for scrutiny and a means of accountability and assurance to communities where research is being conducted, to funders and to the wider public, but it cannot be the only point at which ethics is explicitly considered in the research process. However well executed, ethics committee oversight alone cannot ensure that research is ethical: core responsibilities of other stakeholders within the research ecosystem need explicitly to be recognised and addressed as ethical in ways that are currently widely absent (as evidenced, e.g. in the exploration of funders’ ethical responsibilities embedded within grant allocation processes by Pierson and Millum, 2018, 2022). Even where ethics committees additionally exercise an ongoing audit function after approving a study, this tends to be limited to checking for compliance with the protocol and is not suited to capturing wider or emergent ethical considerations.

This need for a more dispersed approach in exercising ethical responsibilities in research has been long-recognised, at least as far as the responsibility of individual researchers is concerned (Paul, 2018). Indeed, the call to emphasise the role of the ‘intelligent, informed, conscientious, compassionate, responsible investigator’ in ensuring ethical research conduct, for example, goes back to 1966, pre-dating the establishment of current systems of independent review (Beecher, 1966). Responding to the need for research team support in fulfilling this responsibility, there has been growing awareness of the distinction between procedural ethical guidance concerned primarily with documentation and governance and ‘in-practice ethics’ – the way in which researchers respond to those ‘ethically important moments’ that arise in the midst of research practice and which inevitably fall outside the purview of the current ethics review system (Chiumento et al., 2020; Guillemin and Gillam, 2004). It is only more recently, however, that attention has been drawn to the duties and responsibilities of other more powerful actors in the research ecosystem as outlined above, and the importance of characterising these explicitly as ‘ethical’ (Nuffield Council on Bioethics, 2020; Shanks and Paulson, 2022).

## Ethics review in context: Procedural ethics and service delivery

In order to consider how research ethics review systems might evolve to play a valuable role as part of a wider research ethics ecosystem, we need first to recognise how the role of ethics committees has been shaped by the history of medical research abuses that led to their creation (Emanuel et al., 2008; Fischer, 2006). That history has, understandably and rightly, led committees around the world to focus conceptually on the over-riding need to protect participants against possible risk. However, workshop attendees argued that this reaction to the detrimental impacts of exploitative research on individuals has sometimes come at the cost of overlooking issues of equal moral concern relating to social injustice, unequal power relations, exploitation at institution, community or population level, and unfair benefit sharing. In the assessment of risk, it may further lead to a tendency to disregard the harms done through *lack* of research and the consequent inadequate evidence base in many aspects of health (Friesen et al., 2023; Wall and Overton, 2006).

It is also important to recognise how both procedural and substantive responses to these historical abuses, such as the emphasis on the role of expert committees, are themselves shaped by context and culture. Western approaches to substantive ethical deliberation, with their central emphasis on protecting individual autonomy, have had a strong influence in framing ethical deliberation in medicine, including in research ethics committees, in many parts of the world (Chiumento et al., 2020). Yet this narrow focus on the needs of the individual does not necessarily reflect the ethical challenges arising across the globe, especially where research takes place in a historical context of exploitation or oppression, and where critical questions about social justice and whether and how research will benefit wider communities are particularly salient (Hayward et al., 2021; Klitzman, 2013; Sabati, 2019). Moreover, as we indicated earlier, the ‘institutionalisation’ of the systems of ethics review into either national legislation or organisational requirements, risks diverting attention away from what is actually ethically at stake in research and towards simple compliance with regulations, codes, policies and procedures (see Box 1).

Despite the impact of influential guidelines drawing on a wider range of perspectives, such as the 2016 revision of the CIOMS guidelines (Council for International Organizations of Medical Sciences (CIOMS), 2016), these drivers of the ethics review system have helped shape a dominant culture of ‘procedural ethics’ as an adversarial hurdle in research governance, perceived in practice if not in intent as a burden on scientific progress (Hickey et al., 2022). Well-recognised inadequacies in funding, and in the expertise and training necessary to support ethics committee members and staff, put additional pressures on the scope for constructive relationships between researchers and those tasked with reviewing their research (Tusino and Furfaro, 2022). Perhaps inevitably, all these factors have led to critiques of the ethics review system being primarily focussed on what might be described as service provision issues, such as concerns relating to the extent of paperwork, lack of flexibility, speed of response, and redundancy and duplication, considerations made all the more pronounced in the context of reviews of multi-site studies. While these perceptions are not always accurate (see, e.g. the speed of expedited reviews during recent emergencies as documented by, e.g. Palmero et al., 2021; Shekhani et al., 2021), it is nevertheless true that both structural and capacity constraints can lead to slow response times, inconsistent requirements or insufficient expertise to understand what is ethically at stake in particular contexts. As recognised above, this can be exacerbated by the perception on the part of some ethics committees that their role is indeed to ‘police’ research (Klitzman, 2011), in the absence of other ethical checks and balances within the research ecosystem.

There have been, and continue to be, a range of initiatives that seek to address operational concerns on the part of ethics committees, including ongoing work on benchmarking and indicators (Aguilera et al., 2022; World Health Organization (WHO), 2023); training initiatives (Global Health Training Centre, no date; The Global Health Network, no date; Abass, 2017; UNESCO, 2023); and explorations of different ways to optimise the review of complex multi-site studies (Rahimzadeh et al., 2023). Moreover, many ethics committees succeeded in radically transforming their systems in response to the challenges and needs of the COVID-19 pandemic, and are in the process of revising their everyday systems to incorporate some of the procedural innovations and flexibilities developed in response to the exigencies of pandemic research (Wright et al., 2023).

However, it is crucial to distinguish between these questions of how well (or badly) the current systems may work in terms of providing an efficient, timely service to researchers; and the substantive question of the extent to which ethics committee members and staff working within these systems are in a position to grapple with the substantive ethical content of research proposals, and thereby to ensure that this process of scrutiny contributes to the promotion of ‘ethically commendable’ research of scientific *and* social value. Refocusing attention on the value brought to research by ethical scrutiny at this one point in time prompts us to think further about how active ethical engagement and reflexivity can best be incorporated across the *whole* of the research journey – from research question formation to translation of research findings into practice – by all the other stakeholders in research. Reframed in this way, the role of ethics committees becomes one component of a dispersed system promoting, supporting, and verifying ethical research that is contributing to the common good.

## Advocating for a broader view of ethical scrutiny and ethical responsibility

To take a dispersed model forward requires recognition that certain ethical considerations, such as decisions about which communities to engage, which studies to fund, or how researchers respond to unforeseeable challenges in the context of data collection, can only meaningfully be addressed at points in the research process *other* than ethics review, raising the important roles of other stakeholders. A number of organisations and projects have started to introduce systems that facilitate such ethical scrutiny or provide support for researcher reflexivity. We provide some illustrative examples of these in Box 2. However, these kinds of approaches, devised in ways that are sensitive to local contexts, need to become firmly embedded in the practices of *all* research funders, institutions, individual research teams and other relevant actors. This is essential to such approaches becoming an expected and recognised part of the culture of research, rather than aspirational guidelines or exceptional examples of thoughtful practice. Crucially, for meaningful change to be possible in the functions and approaches of ethics committees themselves, committees will need to have assurance that these dispersed responsibilities are routinely recognised, supported, and exercised by other research stakeholders.

## A new starting point: Shifting from the adversarial to the collaborative

What role, then, should ethics committees play in this more distributed system of ethical reflexivity and scrutiny? We believe that the need for ethical scrutiny by a committee independent of the research team should remain as strong as ever, both because of the importance of a transparent system of accountability (to communities where research is taking place, to wider research stakeholders, and to the public), and because of the inherent value of the knowledge and external perspectives that ethics committee members can bring. However, there could and should be important changes, both in the manner and content of that scrutiny.

First, where not already addressed, we highlight the need for the relationship between ethics committees and researchers to be recalibrated so that their interaction is seen as *collaborative* rather than defenive: the exercise of a shared responsibility for ethical research (Connolly and Reid, 2007; Hickey et al., 2022). This certainly does not mean that ethics committees should not scrutinise and, where appropriate, challenge: indeed, it is essential for diverse perspectives to be aired and for researchers to expect to justify their research proposals and designs to others. However, such scrutiny does not need to be exercised in an adversarial spirit. Indeed, an important aspect of an ethics committee’s work should be that of a form of ethical *mentorship*: encouraging ethical reflexivity on the part of researchers and supporting their ability to reflect upon the ethical dilemmas that are bound to arise during their study. In exercising such a role, it will clearly be important for committee members to be alert to the limits of their own knowledge and expertise, and be open to learning in turn from the experience of researchers, topic experts, and wider research stakeholders including those with lived experience.

Second, and more substantively, we suggest that, while retaining a responsibility for the protection of participants, ethics committees should be alert to the dangers of making overly protective, often risk-averse, decisions about others, as captured in often troubling or stigmatising assumptions about vulnerable populations and vulnerability (Bracken-Roche et al., 2017; Greer et al., 2023; Khirikoekkong et al., 2020; Luna, 2019). In particular, we need to challenge the embedded assumption that *not* doing research will always be a safer option, and recognise how sometimes taking what appears to be a precautionary approach or adding in additional safeguards may in practice hinder potentially valuable research without necessarily promoting people’s agency, or protecting them from harm (Nuffield Council on Bioethics, 2015). Overly-protective concerns relating to the inclusion of pregnant women in research, for example, have in practice led to harm through the consequent lack of evidence about the safety and efficacy of drugs during pregnancy (Krubiner and Faden, 2017).

In considering the risks and benefits of a research proposal, we therefore suggest that ethics committees should consider, in particular, how the protocol respects and enhances the agency of the participants alongside protecting their interests, thus treating potential participants as equals in the research endeavour (not solely as ‘others’ to be protected), allowing for a more nuanced approach to the assessment of risk and the minimisation of harm. Importantly, committee scrutiny should also be directed on the extent to which a research proposal contributes towards the common good: sharing responsibility with others, especially funders, in looking at the ‘big picture’ questions of the purpose and value of the research, but with a particular focus on how the proposal is embedded in, and responds to, local needs, contexts and systems.

## Looking forward: Further reflections

We recognise that the approach to the exercise of ethical responsibility and oversight we are putting forward represents a significant change – in the expectations and responsibilities both of ethics committees themselves, and of all other stakeholders within the research ecosystem. Such changes in culture and approach cannot be achieved overnight. However, we suggest that a crucial starting point for a more effective redistribution of responsibilities would be the explicit recognition across the research community that an adversarial, individual-focussed and checklist approach to ethical reflexivity and scrutiny can never be adequate (Nordtug and Haldar, 2024), and that a longitudinal, dispersed approach to the exercise of ethical responsibility must be the aim (and indeed is already being modelled in various ways as described in Box 2). The role of the ethics committee could then be better understood as situated within this wider ecosystem and as one of many actors who hold responsibility for ethically commendable research.

Our proposed shift in the substantive concerns of ethics committees to include consideration of how research protocols can contribute to the common good in ways that promote participant agency, is dependent on culture change elsewhere in the system. This would include, for example, ethical considerations being routinely built into research funders’ processes as described in Box 2; more emphasis on providing support and resources to help researchers in navigating the ethical challenges that arise in practice; and the widespread development of opportunities for early career researchers and postgraduate students to develop their ethical capacities, for example through research ethics discussion forums. In some jurisdictions, change in the remit of ethics committees and their focus of deliberation may also be dependent on amendments to legislation (Ferretti et al., 2021). However, there are also other important ancillary changes that could be implemented more rapidly, and that could help move the dial towards that necessary cultural change across the sector.

Underlying many of our arguments has been the notion of a ‘professional vision’ for ethics committees: the expectation that the work of committees will be conducted effectively and professionally as part of a collaborative endeavour to achieve ethical research conduct and outcomes. Proper **remuneration and recognition** for the time and expertise this requires is a key element of such professionalisation. It cannot be right that this essential part of the research process should commonly rely on voluntary or significantly underpaid labour – and indeed novel ways in which ethics expertise is being embedded into projects, for example through research ethics consultation services or the establishment of ethics and regulatory working groups embedded in trial networks, may already be appropriately compensated (Weinfurt et al., 2017). Where time spent on committee membership is in practice covered by an employer, it is similarly important for the work undertaken to be recognised as being of professional value on a par with other aspects of the person’s role, including, for example, in the context of performance reviews and promotion. On a practical level, it is difficult for members of ethics committees to devote sufficient time (either to scrutinise proposals or participate in training) if that time is unpaid or unrecognised. On the conceptual level, expecting independent ethical scrutiny to be undertaken as unpaid and unrecognised labour sends a clear message as to what is (and what is not) valued, and the place of ethics within wider systems of research.

Another aspect of this professional vision is the recognition that what is asked of ethics committees needs to be **feasible**. Many of the current criticisms of ethics committees (whether relating to service standards such as speed of response, or to the quality of the review such as over-reliance on checklists) arise because people are being asked to do what they are not equipped to do, whether in terms of time, training or support. This reiterates the importance of adequate resources. If the research community is prepared to invest more in its ethics ecosystem – both in a more carefully defined process of ethics review and in the systems necessary to support ethical reflexivity and scrutiny by other stakeholders as described above, then that system will be much better placed to deliver (Wright et al., 2023).

Finally, in thinking about how ethics committees could evolve to provide challenging but collaborative scrutiny within an ethically-responsible research ecosystem, it is crucial to be alert to how well-intentioned systems and procedures (designed to operationalise particular values-based approaches) can themselves become inflexible and bureaucratic (Strathern, 2000). There is no easy answer to this conundrum – systems and procedures will always be necessary – but it highlights again the importance of committee members, chairs and staff who have the skills and confidence to use, but not be constrained by those systems. Ultimately, we seek to promote an approach that keeps in view the values ethics committees are striving to maintain, and that supports them in exercising reflexivity in the way they fulfil their own roles.

## Conclusion

We have argued that the ethical conduct of research is the responsibility of *all* stakeholders in the research ecosystem – from funders, governments and research institutions to individual research teams and ethics committees. As a research community we need to recognise the rapidly evolving nature of research in the health sector, and the expansion of substantive ethical engagement around questions of the promotion of the ‘common good’ through health research more broadly. In order to attain what we have termed ‘ethically commendable research’, we call on the research community to espouse, and take action to achieve, more distributed approaches to ethical scrutiny and reflexivity. We propose that crucial elements of such a shift should be the development of collaborative relationships between researchers and ethics committees that recognise and respect the mutual responsibilities of all parties to promote ethical research conduct. The examples highlighted aim to provide illustrations of how dispersed ethical responsibilities are being engaged with across research in the health sector, offering models for others to take up and adapt to their contexts. In tandem with these elements, we identify some ways in which ethics committees may need to reconceptualise their role to recognise formal ethical review as providing one pillar of the ethical oversight of research which promotes the common good.

## Footnotes

1. The majority of whom are also co-authors.

2. Known as Research Ethics Committees (RECs) or Institutional Review Boards (IRB) depending on jurisdiction; referred to collectively in this article as ethics committees.
